# Involvement of Ceramide Signalling in Radiation-Induced Tumour Vascular Effects and Vascular-Targeted Therapy

**DOI:** 10.3390/ijms23126671

**Published:** 2022-06-15

**Authors:** Deepa Sharma, Gregory J. Czarnota

**Affiliations:** 1 Physical Sciences, Sunnybrook Health Sciences Centre, Toronto, ON M4N 3M5, Canada; gregory.czarnota@sunnybrook.ca; 2 Department of Radiation Oncology, Sunnybrook Health Sciences Centre, Toronto, ON M4N 3M5, Canada; 3 Departments of Medical Biophysics and Radiation Oncology, University of Toronto, Toronto, ON M4N 3M5, Canada

**Keywords:** apoptosis, cell death, ceramide, endothelial cell, ultrasound-stimulated microbubbles, vascular damage

## Abstract

Sphingolipids are well-recognized critical components in several biological processes. Ceramides constitute a class of sphingolipid metabolites that are involved in important signal transduction pathways that play key roles in determining the fate of cells to survive or die. Ceramide accumulated in cells causes apoptosis; however, ceramide metabolized to sphingosine promotes cell survival and angiogenesis. Studies suggest that vascular-targeted therapies increase endothelial cell ceramide resulting in apoptosis that leads to tumour cure. Specifically, ultrasound-stimulated microbubbles (USMB) used as vascular disrupting agents can perturb endothelial cells, eliciting acid sphingomyelinase (ASMase) activation accompanied by ceramide release. This phenomenon results in endothelial cell death and vascular collapse and is synergistic with other antitumour treatments such as radiation. In contrast, blocking the generation of ceramide using multiple approaches, including the conversion of ceramide to sphingosine-1-phosphate (S1P), abrogates this process. The ceramide-based cell survival “rheostat” between these opposing signalling metabolites is essential in the mechanotransductive vascular targeting following USMB treatment. In this review, we aim to summarize the past and latest findings on ceramide-based vascular-targeted strategies, including novel mechanotransductive methodologies.

## 1. Introduction

### 1.1. ASMase and Ceramide

Acid sphingomyelinase (ASMase, EC 3.1.4.12) is a key enzyme in the sphingolipid metabolic pathway [1,2]. The involvement of the ASMase enzyme in disease states was first reported in people suffering from autosomal recessive genetic disorders known as Niemann–Pick disease types A and B [3]. Excessive accumulation of sphingomyelin was noted with these disorders. There are two main types of ASMase—namely lysosomal acid sphingomyelinase (L-SMase), known to be zinc (Zn(2+))-independent, and a secreted acid sphingomyelinase (S-SMase) that is zinc (Zn(2+))-dependent [3,4,5,6]. Both types differ in their trafficking process, utilizing a common protein precursor, ASMase. L-SMase, which is localized in an endolysosomal compartment, is translocated to an extracellular leaflet through the fusion of the lysosome to the plasma membrane for secretion [7,8]. S-Mase, on the other hand, is released extracellularly through the Golgi pathway.

**Figure 1:**
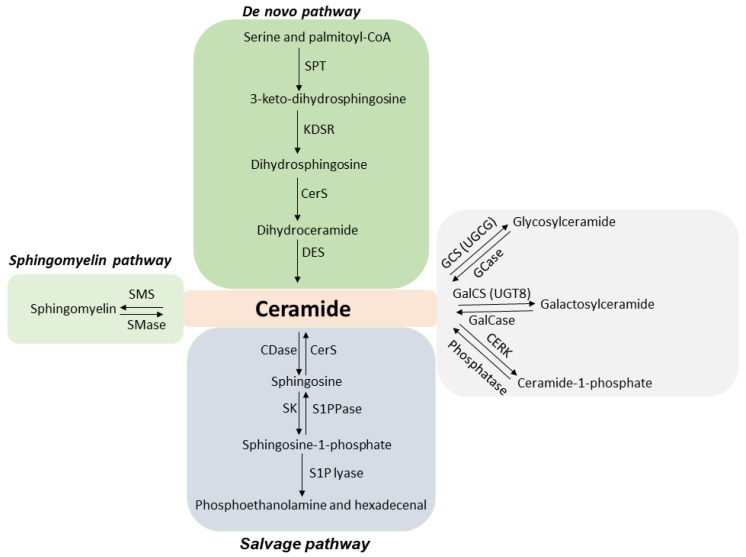
Diagram depicting ceramide synthesis and metabolism pathways. Ceramide generation takes place by three different pathways. The first one occurs at the plasma membrane in the presence of enzyme acid sphingomyelinase (ASMase). Hydrolysis of sphingomyelin in the presence of ASMase results in ceramide release. Secondly, the de novo pathway regulates ceramide generation by the condensation of palmitate and serine. 3-keto-dihydrosphingosine formed through this process is catabolized further to dihydroceramide. Ceramide generated in the endoplasmic reticulum is transported to the Golgi apparatus. This process is mediated by vesicle trafficking or by the involvement of ceramide transfer protein (CERT). Lastly, ceramide is generated by sphingolipids and glycosphingolipids degradation through the salvage pathway taking place in the late endosomes and the lysosomes. Ceramide can also be converted to complex sphingolipids. Abbreviations: CDase—ceramidase; CERK—ceramide kinase; CerS—ceramide synthase; DES—dihydroceramide desaturase; GalCase—galactosylceramidase; GalCS—galactosylceramide synthase; GCS—glucosylceramide synthase; GCase—glucocerebrosidase; KDSR—3-ketodihydrosphingosine reductase; S1P lyase—sphingosine-1-phosphate lyase; S1PPase—sphingosine-1-phosphate phosphatase; SK—sphingosine kinase; SMase—sphingomyelinase; SMS—sphingomyelin synthase; SPT—serine-palmitoyl transferase; UGCG—UDP-glucose ceramide glycosyltransferase; UGT8—UDP glycosyltransferase 8.

**Figure 2:**
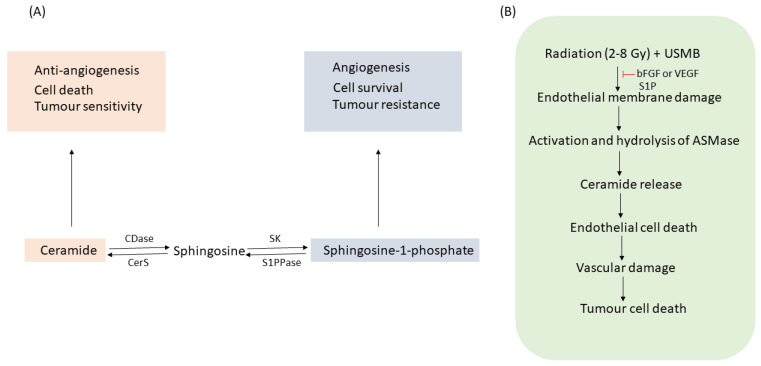
(**A**) The ceramide and S1P rheostat in the regulation of cell death and survival. An increase in ceramide inhibits angiogenesis, making cells sensitive to radiation therapy. On the other hand, S1P elevation causes cells to proliferate, promoting angiogenesis and resulting in resistance to radiotherapy. (**B**) Effect of radiation and USMB on tumour damage. Radiation dose (2–8 Gy) combined with USMB induces ASMase/ceramide activation. This leads to endothelial membrane perforation causing vasculature damage, ultimately killing tumour cells. The entire process can be abrogated using bFGF/VEGF or ceramide antagonist, S1P. Abbreviations: ASMase—acid sphingomyelinase; bFGF—basic fibroblast growth factor; CDase—ceramidase; CerS—ceramide synthase; S1PPase—sphingosine-1-phosphate phosphatase; SK—sphingosine kinase—S1P—sphingosine-1-phosphate; USMB—ultrasound-stimulated microbubbles; VEGF—vascular endothelial growth factor.

ASMase is known to have various functions, one of which includes facilitating the synthesis of ceramide, a cell death molecule. Ceramide is a bioactive lipid molecule comprised of sphingosine and a fatty acid. Ceramide generation takes place by three different pathways (Figure 1): the sphingomyelinase hydrolysis pathway, the de novo pathway, and the salvage pathway [1,9,10,11,12]. The first pathway involves the hydrolysis of sphingomyelin in the presence of the ASMase enzyme, resulting in ceramide generation. The process takes place in the cell plasma membrane. Ceramide synthesis via de novo pathways initiates by the condensation of serine and palmitoyl-CoA generating 3-keto-dihydrosphingosine in the presence of enzyme serine-palmitoyl transferase (SPT). 3-keto-dihydrosphingosine reductase then regulates the generation of dihydrosphingosine, which is acetylated by ceramide synthase (CerS), forming ceramide. Ceramide synthesis by this pathway takes place in the endoplasmic reticulum, followed by its transportation to the Golgi apparatus. Ceramide produced through the salvage pathway is initiated through the degradation of sphingolipids and glycosphingolipids [9]. This process takes place in late endosomes and lysosomes [13]. Ceramide in this pathway is degraded to sphingosine by ceramidase (CDase). Sphingosine can also be recycled back to ceramide by ceramide synthase (CerS). Sphingosine is then phosphorylated to sphingosine-1-phosphate (S1P) by sphingosine kinase (SK). S1P can be dephosphorylated to sphingosine by S1P phosphatase (S1PPase) or degraded by S1P lyase to phosphoethanolamine and hexadecenal. Ceramide once formed can be metabolized to other bioactive sphingolipids such as phosphorylation of ceramide by ceramide kinase (CERK) resulting in the synthesis of ceramide-1-phosphate (C1P). Ceramide can also be converted to glucosylceramide or galactosylceramide by glucosylceramide synthase (GCS)/UDP-glucose ceramide glucosyltransferase (UGCG) or galactosylceramide synthase (GalCS)/UDP galactosyltransferase (UGT8), respectively. Ceramide generated through all three pathways has been reported to have an important role in cell signalling processes [14,15,16,17,18,19]; however, the role of ceramide signalling generated through the activation of ASMase has been explored and studied the most. Exposure to various stress stimuli, including anticancer treatments, causes ASMase to translocate from lysosomes to the cell plasma membrane [20,21,22,23]. Once translocated, ASMase induces the hydrolysis of sphingomyelin to generate ceramide [24]. Ceramide, when formed, is a small molecule that self-associates to form ceramide-enriched microdomains. These microdomains fuse further to form ceramide-enriched macro domains/platforms [25,26,27,28]. These platforms trap and cluster several signalling molecules and receptors, activating signal transduction pathways [27,29,30,31,32,33,34]. Ceramide then acts as a second messenger, facilitating the activation of several apoptotic cascades [16,35,36]. Accumulation of ceramide on cancer cells promotes cell death and inhibits tumour survival and angiogenesis [14,37,38]. In contrast, ceramide degraded to its metabolite S1P (ceramide antagonist) induces cancer cell survival, proliferation, and angiogenesis [39,40,41,42]. Thus, a balance between ceramide and S1P is known to be a determining factor for cells to die or survive (Figure 2A). Several studies have utilized the approach of combining ceramide or its metabolites with antiangiogenic therapy or vascular targeting therapies for better outcomes [43,44]. Inducing ceramide using radiotherapy or chemotherapy combined with antiangiogenic inhibitors or vascular targeting agents is proven to be beneficial in curing various cancers in preclinical models [45,46,47]; however, understanding the role of ceramide and its metabolites in the improvement of overall survival in patients with advanced solid tumours following cancer therapy is still an ongoing process.

### 1.2. Microbubbles and Ultrasound: Ultrasound-Stimulated Microbubbles

**Figure 3:**
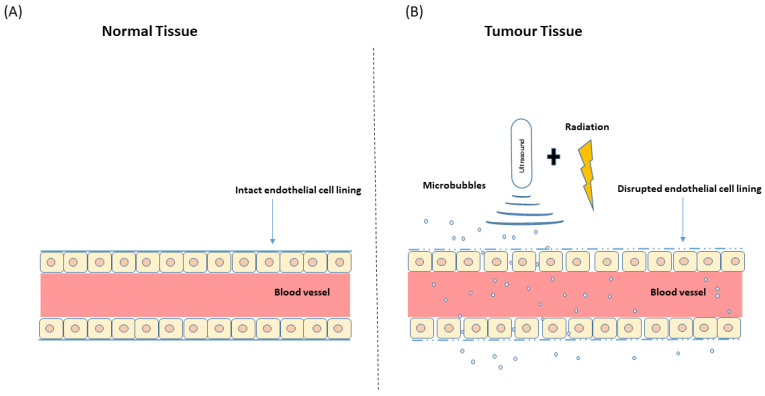
Illustration of normal and tumour vasculature. (**A**) Normal blood vessels are highly organized with regular intact endothelial lining. (**B**) Tumour blood vessels are irregularly shaped with the loosely associated basement membrane. USMB and radiation-induced endothelial membrane perforation result in enhanced vascular permeability inducing greater tumour response. Abbreviations: USMB—ultrasound-stimulated microbubbles.

Microbubbles are tiny gas-filled bubbles less than 10 µm in diameter that are stabilized within a shell composed of lipid, protein, or polymers [48,49,50,51]. The gas in microbubbles is either air, nitrogen, perfluorocarbon, or sulfur hexafluoride [48,52,53]. The shell of a microbubble is known to affect its physical properties, including elasticity, compressibility, stability, and response to acoustic energy. Microbubbles are inert under stable conditions and pass through the human body, eventually decaying, with a half-life typically less than 5–10 min [52,53]. The small size and the acoustic impedance of the gas–fluid interface make microbubbles excellent contrast agents. Specifically, when placed under an acoustic beam at a resonant frequency, the gas core of the bubbles responds to the intensity of the acoustic wave by compression and rarefaction. Low-intensity ultrasound beams cause bubbles to undergo compression and rarefaction cycles without bursting and can be stimulated over longer periods while maintaining the structural integrity of these bubbles. This is known as stable cavitation. In contrast, inertial cavitation takes place when high-intensity ultrasound beams act upon bubbles, causing them to oscillate chaotically and sometimes causing them to reach beyond a maximum diameter, resulting in bubble destruction [50,54]. Many reviews go further in-depth to describe different properties and applications of microbubbles [51,55,56,57]. The destruction of microbubbles close to cell surfaces induces perforations in the plasma membrane through a process known as sonoporation. This process permits the entry of substances subsequently across the endothelial lining of blood vessels (Figure 3). The holes in the cell membrane can vary from the size of a nanometer to a micrometer depending on the size of the microbubbles interacting with the cell membrane, and this can alter the permeability of the plasma membrane. These holes generate a cellular response to reseal them, and if the damage induced by the ultrasound-stimulated microbubbles (USMB) is sufficiently large, the cells will die as a result of their microbubble-induced injuries [58,59]. The effects of USMB on tumour vasculature have been shown to facilitate an enhanced sensitivity towards chemotherapy [60,61,62] and radiation therapy (XRT) [63,64,65]. The safety of using USMB in clinical settings has already been established for the treatment of several diseases; however, the idea of utilizing USMB as a therapeutic agent for cancer has only recently been looked into. Currently, research has focused on USMB treatment-enhancing properties in drug delivery and radiotherapy. Although most studies conducted are preclinical, a number of clinical studies have been carried out in order to determine the safety of USMB therapy in patients with different diseases [66,67,68,69,70]. USMB combined with chemotherapy and or XRT has been explored extensively in preclinical models and has succeeded in exhibiting fruitful outcomes. Although there have been few clinical and several preclinical studies on microbubble-based chemotherapy using brain tumour demonstrating outstanding antitumour effectiveness, not much has been explored in treating brain tumours with USMB combined with XRT. Most of the studies dealt with the feasibility of USMB; however, recently an attempt has been made by Prada and colleagues to quantify microbubble distribution in the human brain with glioblastoma. The highest degree of microbubble enhancement was reported to be within arteries, followed by the tumour area and then the white matter [71]. Thus, this first attempt to quantify cerebral microbubble distribution and mapping microbubbles within the brain area will be advantageous in the future for understanding the target points of ultrasound to treat patients with ultrasound-microbubble therapy. Since whole-brain radiation therapy or stereotactic radiosurgery has already been established for treating brain tumours, incorporating ultrasound-microbubble therapy along with XRT might enhance the effectiveness of XRT in treating malignant brain tumours.

### 1.3. Radiation-Induced Cell Death

For many years, it was believed that DNA was the major target in radiation-induced cell death. Ionizing radiation generates lethal lesions in cellular DNA, causing cell-cycle linked cell death [72,73,74]. An alternative mechanism to this was provided by Fuks and Kolesnick, suggesting the cell plasma membrane as a crucial target in radiation-based cell kill [75,76,77]. Their work indicated that radiation-induced alterations in the cellular membrane initiate the activation and hydrolysis of sphingomyelin, leading to ceramide release. Ceramide, once generated, acts as a proapoptotic second messenger that leads to the activation of a cell-death signalling cascade. Haimovitz-Friedman et al. first reported in their study that exposure of bovine aortic endothelial cells (BAEC) to radiation caused membrane ASMase-linked ceramide production, activating an apoptotic pathway. This was validated using cells that were free of nuclei, confirming the exclusion of DNA damage for initiating cell death [24]. Two separate and distinct responses of ceramide generation are reported upon irradiation. First is a transient ceramide increase, which is known to be ASMase dependent but DNA-damage independent [78]. A second wave of DNA damage-induced ceramide increase is reported to take place much later (after several hours) of irradiation that is mainly regulated by the involvement of ceramide synthase [79]. It has been reported that ionizing radiation-induced ceramide-based cell death is both dose and time-dependent, and this has been validated in several in vitro and in vivo studies [76,80]. The mechanism of radiation-induced cell death is variant, depending on a low or high dose of ionizing radiation. Cell death induced after a single high dose of radiation (>8–10 Gy) is known to be governed by the ASMase-ceramide pathway; however, the mechanism of cell kill upon low dose exposure is dependent on reactive oxygen species (ROS) production; however, a common phenomenon observed with either of the doses includes endothelial cell damage. Moeller et al. discovered that exposure to fractionated doses (1.8–3 Gy) leads to DNA damage and additionally an activation/upregulation of hypoxia-inducible factor (HIF)-1 and generation of ROS. ROS release causes translation of HIF-1 mRNA, accompanied by the production of proangiogenic growth factors. This leads to endothelial apoptosis concomitant with tumour cell death and overall enhancement in tumour response [81,82]. Several studies have now confirmed that combining antiangiogenic or vascular disrupting agents with low dose irradiation mimics the endothelial vascular damage induced upon a single high dose. Extensive work by Czarnota et al. also shows that USMB combined with low-dose irradiation mimics similar effects caused by a single high dose of radiation. USMB and radiation cause endothelial membrane perturbation, leading to ASMase-dependent ceramide release. This further causes endothelial damage and vascular collapse, subsequently resulting in tumour cell death (Figure 2B). Potential tumour growth inhibition using this technique has been confirmed in several in vitro and in vivo works [47,65,83,84].

The present review provides an insight into the use of USMB in combination with radiation to enhance overall tumour response. Further, the biological effects of USMB, including cell death and vascular effects, will be discussed in detail. The review will also discuss the mechanism involved in mechanical endothelial perturbation that occurs when USMB therapy is combined with low radiation doses. Lastly, the review will highlight the clinical relevance of ceramide and the perspective of using USMB as a vascular targeting agent to enhance the effects of XRT.

## 2. Microbubble-Based Radiation Enhancement: Ceramide-Induced Cell Death

### In Vitro Studies

**Figure 4:**
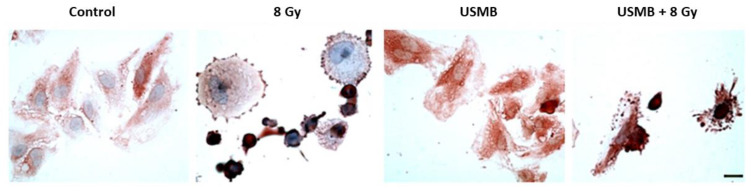
Ceramide immunohistochemical staining in HUVEC treated with USMB, XRT, and USMB + XRT. Cells were treated with XRT alone, USMB alone, and a combination of USMB and 8 Gy. Images captured at high magnification indicate higher ceramide staining in the combined treated group of USMB and 8 Gy. Ceramide stains were also noted in the radiation-only group of 8 Gy; however, the staining was less intense compared to combined treatment. Control group cells appeared to have lesser ceramide staining. The scale bar represents 25 µm. Abbreviations: HUVEC—human umbilical vein endothelial cells; USMB—ultrasound-stimulated microbubbles; XRT—radiation therapy. Adapted with permission from [87].

**Figure 5:**
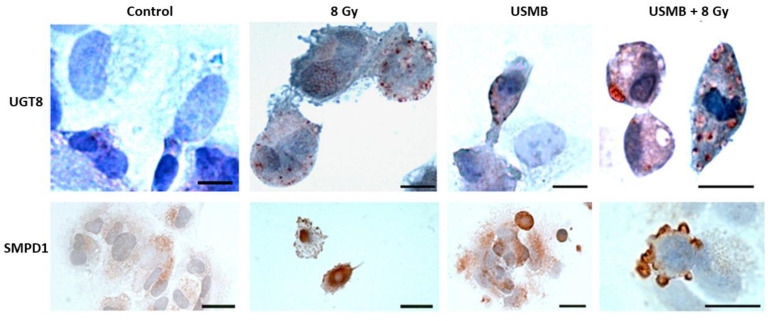
UGT8 and SMPD1 immunohistochemistry of HUVEC following different conditions. The upper panel images depict UGT8 and the lower panel represents SMPD1 following various treatments. Both UGT8 and SMPD1 stains were higher in the combined treated group. Cells appeared to have distorted morphology, indicating apoptosis-like features in both UGT8 and SMPD1 combined treated group. Scale bar represents 25 µm for control, 8 Gy, and USMB and 12 µm for USMB + 8 Gy. Abbreviations: HUVEC—human umbilical vein endothelial cells; SMPD1—sphingomyelin phosphodiesterase 1; UGT8—UDP glycosyltransferase 8; USMB—ultrasound-stimulated microbubbles. Adapted with permission from [87].

Ceramide-induced cell death has been confirmed in several in vitro and in vivo studies. This section compiles a selection of in vitro work emphasizing the importance of ceramide in activating the cell death cascade and enhancing the overall tumour response. An early study by Nofiele et al. evaluated the involvement of ceramide in cancer cell death following USMB and radiation treatment. Five different cell types were included in experiments, namely human umbilical vein endothelial cells (HUVEC), acute myeloid leukemia cells (AML), murine fibrosarcoma cells (KHT-C), prostate cancer cells (PC3), breast cancer cells (MDA-MB-231), and astrocytes (ASMase +/+) and (ASMase −/−). Survival rates and changes in cellular morphology were confirmed using clonogenic assays and immunohistochemistry analysis, respectively. Immunohistochemical staining of cells was performed to visualize ceramide content. Hematoxylin and eosin (H&E) staining confirmed prominent cellular shrinkage and morphological structural changes such as nuclear condensation and fragmentation upon exposure to USMB treatment. A higher accumulation of ceramide was observed in cells treated with USMB and radiation compared to untreated cells or those receiving individual treatments. Furthermore, survival assays were performed with HUVEC and ASMase +/+, ASMase −/− astrocytes. The selection of these two types of cell types was made to validate the involvement of ASMase-induced ceramide signal transduction. HUVEC cells are known to have an enhanced level of ASMase. The survival rate in these cells was found to be significantly lower following USMB and radiation; however, the addition of S1P resulted in an increased survival rate in HUVEC cells. A similar response in cell death was reported for ASMase +/+, ASMase −/−, and S1P-treated astrocytes [85]. Later, Al-Mahrouki et al. investigated the molecular mechanisms linked with ceramide signalling that cause endothelial cell death. For this, HUVEC cells were exposed to USMB alone, XRT alone, and a combination of USMB + XRT. Treatment effects were evaluated after 0, 3, 6, and 24 h. A greater extent of morphological alterations such as nuclear pyknosis, bi-nucleation, and apoptotic body formation was observed in endothelial cells following USMB + XRT treatment. Ceramide staining in cells exposed to USMB + XRT was higher than that in untreated cells or cells treated with USMB or XRT alone (Figure 4). The level of ceramide was found to be highest at 6 h, after which ceramide staining was less apparent. Furthermore, exogenous ceramide (C2) and several ceramide antagonists such as fumonisin B1, monensin, and S1P were used to evaluate the survival efficacy of endothelial cells. Incubating the cells with exogenous ceramide demonstrated a significant reduction in survival rates, in contrast to using ceramide inhibitors that enhanced cellular survival. Moreover, immunofluorescence staining detected the co-localization of ceramide modulator with mitochondria, suggesting the involvement of both ASMase, and de novo, synthesized ceramide release in causing cell death [86]. For many years, several strategies have been developed targeting ceramide metabolism, either by using ceramide agonists or antagonists; however, very few attempts have been made to discover the genetic variation at the molecular level that governs the up and down-regulation of ceramide. One of the previous studies conducted by Al-Mahrouki et al. revealed that genes such as sphingomyelin phosphodiesterase 2 (SMPD2), UDP glycosyltransferase 8 (UGT8), cytochrome c oxidase subunit 6B1 (COX6B1), Caspase 9, and mitogen-activated protein kinase 1 (MAP2K1) were found to be up-regulated with the exposure of endothelial cells to USMB (Figure 5). These genes are known to be involved in ceramide-mediated apoptotic pathways [87]. A few years later, the role and regulation of the UGT8 gene in mediating ceramide-based signalling pathway were evaluated in detail. For this, genetically modified PC3 cells were exposed to USMB, XRT, or a combination of USMB and XRT. Results revealed that UGT8 down-regulated cells accumulated ceramide, demonstrating a significant reduction in cell survival as compared to control and up-regulated groups. The study further evaluated tumour response in vivo using PC3 tumours xenograft in which UGT8 was up and down-regulated [88] (this is explained in detail in the section below).

## 3. Microbubble-Based Radiation Enhancement: Ceramide-Induced Cell Death

### In Vivo Studies

**Figure 6:**
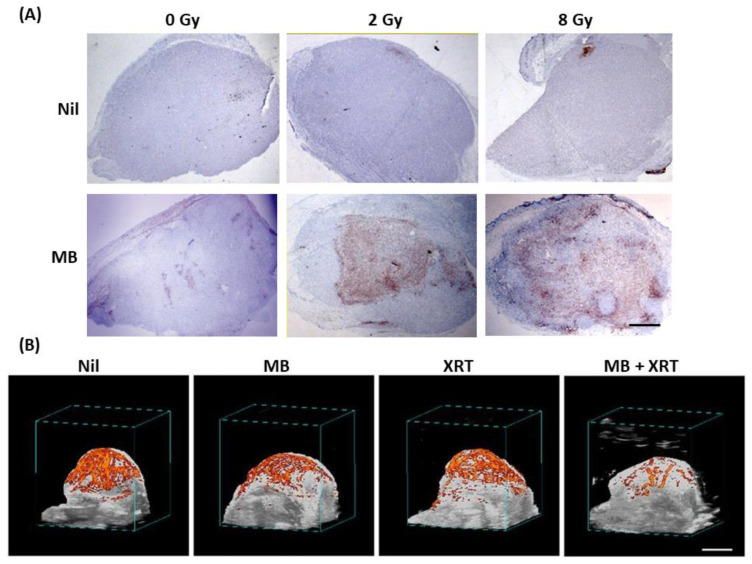
In vivo cell death staining of sections of PC3 prostate tumours and power Doppler ultrasound imaging for radiation and ultrasound treatments. (**A**) Image depicts assessments of cell death in PC3 xenografts following various concentrations of microbubbles and different radiation doses. Microbubbles stimulated at 570 kPa combined with XRT demonstrated prominent cell death compared to the one with no USMB. Scale bar: 2 mm. (**B**) On the contrary, tumour blood flow determined using 3D power Doppler ultrasound revealed lower blood flow with the combined treatment of USMB and XRT, whereas the control, USMB alone, and XRT alone showed higher blood flow. Scale bar: 2 mm. Abbreviations: MB—microbubbles; USMB—ultrasound-stimulated microbubbles; XRT—radiation therapy. Adapted with permission from [65].

The process of endothelial cell death is one of the main and essential contributing factors to tumour vasculature damage. ASMase-linked ceramide mediates cell death in many cell types, including endothelial cells. Endothelial cells exhibit 20% greater ASMase compared to any other cells, making them a potential target when using ASMase/ceramide-based treatment. A study carried out by Czarnota et al. discussed ceramide-induced endothelial and vascular damage in detail [65]. Effects of different treatment parameters, including different microbubble concentrations (up to 100-fold dilutions), different radiation doses (0, 2, and 8 Gy), and different treatment durations (0, 3, 6, 12, and 24 h) were explored. More than 300 PC3 prostate cancer xenograft-bearing mice were included in that study. Results demonstrated significant cell death when USMB was combined with XRT. Minimal cell death of (mean ± SE) (10 ± 4%) and (4 ± 2%) was observed with USMB and 2 Gy alone, respectively. The combination of 2 Gy with USMB resulted in (44 ± 13%) cell death. The greatest cell death (70 ± 8%) was observed with USMB combined with 8 Gy (Figure 6). Furthermore, a combination of USMB and XRT resulted in endothelial cell apoptosis that was confirmed using triple staining of tumour section that included 4′,6-diamidino-2-phenylindole (DAPI) for nuclei, cluster of differentiation 31 (CD31) for viable endothelial cells and terminal deoxynucleotidyl transferase dUTP nick end labeling (TUNEL) for detecting DNA fragmentation. The optimal time that depicted the maximum amount of cell death was reported to be 6 h. Cell death was found to be similar or declined after this time. The study further revealed that tumour exposed to combined treatment of USMB and XRT resulted in significant blood flow diminishment, conferring vascular deterioration. The involvement of ceramide in inducing cell death and vessel damage was confirmed using ceramide and S1P staining. A combination of USMB and 8 Gy demonstrated greater than 60% ceramide staining, compared to radiation only with 32%. Tumour sections stained with S1P demonstrated lesser ceramide staining concomitantly with less apoptotic cell death, confirmed with H&E and in situ end-labeling (ISEL) staining. Thus, the study confirmed that ceramide generated in response to the disruption of USMB and XRT exposure contributed to endothelial apoptosis and vascular damage [65]. These findings were further validated in several other xenograft models [83,84,89,90].

Another study by Kim et al. using PC3 xenograft reported that ceramide is generated in both endothelial and tumour cells, which was found to enhance as the concentration of microbubble and radiation dose were increased [90]. Recently, El Kaffas et al. undertook a genetic approach to validate the aforementioned findings using ASMase+/+ wild type (WT), −/− knockout (KO), and S1P-WT treated mice implanted with fibrosarcoma xenografts. Tumours treated with USMB and radiation demonstrated greater vasculature deterioration followed by extensive tumour cell death in WT xenograft compared to S1P treated or knockout groups. Furthermore, triple staining using ceramide, CD31, and DAPI was performed to detect the ceramide content and the presence of endothelial cells and cell nuclei. WT mice treated with USMB + 8 Gy, at 24 h, demonstrated the highest prominent ceramide staining, whereas S1P-treated or ASMase KO mice depicted lesser stains. The level of various other ceramide species, which would be formed by ASMase, ceramide kinase (CERK), and ceramide synthase 1 (CS1), were detected using mass spectrometry in all three grouped animals. Assays were also performed to detect these enzyme levels. Although ASMase and CS1 induce cell death, CERK, on the other hand, promotes cell survival and proliferation. Elevated ASMase and CS1 content were found in the WT tumour section following combined USMB and XRT compared to S1P and KO groups; however, the level of CERK was found to be enhanced significantly only in the WT group [47]. Thus, their study confirmed the involvement of the ceramide pathway in mediating endothelial apoptosis followed by vascular damage and tumour cell death.

At present, ceramide and various related lipid-biogenesis pathways are potential targets to initiate endothelial vascular damage to improve overall tumour response. One of the species that has widely been studied is UGT8, which is specifically involved in lipid biogenesis. Al-Mahrouki et al. explored the ceramide-based cell death pathway by up and down-regulating the UGT8 gene. Their study included both in vitro and in vivo PC3 findings that suggested accumulation of ceramide in the down-regulated model depicted greater cell damage with lesser cellular survival rates. For up-regulation of UGT8 in an in vitro model, PC3 cells were stably transfected with the coding sequence of UGT8, and for the in vivo work, xenograft tumours were generated by injecting the stably transfected PC3 cells in the hind leg of male mice. Results from in vitro work depicted up-regulated UGT8 to have lesser ceramide staining and greater cellular survival level followed USMB + 8 Gy compared to down-regulated UGT8, which marked more ceramide content and reduction in cell survival. Similarly, H&E, TUNEL, and ceramide labelling were performed with the xenograft sections to evaluate treatment response. H&E and TUNEL stained sections showed greater cellular damage, with cells mostly undergoing apoptosis, necrosis, and fibrosis in the down-regulated group. The quantified staining of the tumour section with TUNEL revealed the highest level of cell death in a down-regulated group compared to control and up-regulated groups. Similarly, a significant increase in ceramide staining was observed in the down-regulated model compared to control or UGT8 up-regulated tumours. Furthermore, the level of tumour blood flow and oxygen saturation were also found to be attenuated in the down-regulated group following USMB and XRT. These data indicated the importance of UGT8 gene expression in the tumour cells, suggesting ceramide as a pivotal player in causing an endothelial vascular collapse that subsequently leads to tumour cell death [88].

## 4. USMB and Radiation Effect on Tumour Vasculature

The functioning of endothelial cells is important for the maintenance of vascular integrity and proper blood flow. An adequate supply of blood is as essential for cancerous tissue as it is for its normal counterparts. Normal tissue exhibits well-organized blood vessels consisting of arterioles, capillaries, and venules. In contrast, tumour vasculature contains irregular branches that are tortuous and leaky [91,92]. Due to their abnormal structure, blood flow in tumours tends to be significantly lower and hence acquiring sufficient blood supply becomes of utmost importance [93]. Lack of nutrients such as blood and oxygen supply makes tumours resistant to many therapies, which make treatment difficult, resulting in therapy failure [94]; therefore, determining tumour blood flow and oxygen content helps to understand the tumour physiology in a better way. Numerous studies have confirmed that blood flow is a governing factor of tumour response to radiotherapy [47,84]. These findings have shown that there exists a direct correlation between endothelial damage and vascular shutdown with tumour cell death. This process starts with endothelial cell apoptosis followed by vascular collapse leading to secondary tumour cell death and the phenomenon has been validated using several in vitro and in vivo models [65,88,95,96]. Most of the studies utilized the conventional histological method to detect cell death both in endothelial and in tumour cells, whereas for blood flow detection, three-dimensional (3D) Doppler ultrasounds have been used. One of the studies measured blood flow in PC3 xenograft of mice using 3D power Doppler ultrasound. Tumours exposed to different treatment conditions of USMB, XRT, and a combination of USMB and XRT were evaluated for blood flow content at 0, 3, 6, 12, and 24 h. The result demonstrated a significant reduction in a power Doppler-measured vascular index at 24 h, indicating (mean ± standard error) a drop to 65 ± 8% for the combined USMB and XRT treatment compared to 20 ± 21% and 20 ± 32% for USMB and 8 Gy, respectively, compared to before treatment. Cell death was linked to decreases in vascular index, with the highest apoptotic index for a combination of USMB and XRT compared to USMB alone and radiation alone. Thus, their findings suggested that tumours exposed to a combination of USMB and XRT exhibited maximum cell death that coincided with minimal tumour blood flow [65] (Figure 6). Briggs et al. reported similar findings in which PC3 xenograft tumour, when exposed to USMB and XRT, demonstrated a decrease in oxygen saturation and blood flow measured with photoacoustics imaging technique and power Doppler, respectively. Furthermore, histological assessment of tumour sections revealed an increase in cell death and a decrease in intact vasculature confirmed using ISEL and CD31 staining, respectively, with identical treatments. The study suggested a correlation that existed between oxygen saturation and blood flow decrease to that of cell death increase and reduced intact vasculature [95]. PC3 xenograft model is known to have enhanced ceramide levels upon exposure to USMB and XRT [90]. A novel mechanism was proposed on the PC3 model indicating a combination of USMB and XRT exposure induces ceramide production that causes reactive oxygen species to release further, leading to vasculature damage and secondary tumour cell death [89]. Thus, the entire process of vascular disruption and tumour cell death is reported to be ceramide dependent.

The dependence of ceramide in endothelial damage and dysfunctioning of blood vessels has also been explored using ASMase+/+ (WT), ASMase−/− (KO), and S1P-treated WT C57BL/6 mice. Blood flow and cell death in those mice treated with different combinations of USMB and radiation were extensively explored. Blood perfusion measured with power Doppler in WT tumours treated with a combination of USMB and XRT was found to be significantly lower, indicating a decrease of 46.5% observed at 3 h, subsequently peaking at 24–72 h. An opposite trend with an increase in cell death was observed in the same group. On the contrary, changes in blood perfusion and tumour cell death were found to be minimal in the S1P-treated and KO group with similar treatments of USMB and XRT. The study further reported that ceramide level was found to be increased in WT mice receiving combined treatment, while minimal ceramide content was demonstrated in S1P and KO mice [47]. The study concluded ceramide as a major contributing factor for tumour vascular shutdown leading to cell death following USMB and radiation. Endothelial radiosensitization involving the ASMase-ceramide pathway was reported to be achieved either by using a single high dose (>8–10 Gy) or low radiation doses combined with USMB. Thus, the results from the abovementioned experimental tumours strongly indicate that USMB and radiation act together in activating the ASMase-ceramide pathway, thereby acutely disrupting the tumour perfusion leading to intratumour microenvironment damage and indirect tumour cell death.

## 5. Effects of Sequencing and Treatment Parameters

Most of the preclinical studies have usually administered USMB prior to radiation. Very few have explored reversing the sequence. Klein et al. in their study, explored the order and timing of these treatments on treatment response. Firstly, USMB was followed by XRT (USMB-XRT) and secondly, XRT was followed by USMB (XRT-USMB). For the experiments, prostate cancer xenografts were exposed to USMB and XRT. The treatment gap between USMB and XRT was separated by 0, 3, 6, 12, and 24 h. Different histological analyses, including H&E, TUNEL, CD31, and CA-9 staining, were used to assess treatment effects. The study reported maximum effect when USMB was administered before XRT (USMB-XRT) and the time interval between these two treatments was 6 h. H&E staining confirmed greater changes in cell nuclei and a higher number of apoptotic cells at 6 h in the combined treated group, irrespective of sequence (USMB-XRT or XRT-USMB). Cell death detected with TUNEL for the USMB-XRT treated group at 0 and 6 h was reported to be (mean ± standard deviation) 19% ± 4%, and 36% ± 10%, respectively. Maximum reduction in microvessel density was observed at 6 h, which coincident with the highest cell death observed at the same time point (6 h). Similarly, the hypoxia level was found to be maximum for USMB-XRT and XRT-USMB at 6 h compared to USMB-XRT and XRT-USMB, respectively, at 0 h [97]. This study looked at the effect of reversing the sequence for USMB and XRT; however, the parameters for each treatment condition were kept constant. Kim et al. conducted a study to evaluate the effect of several permutations using PC3 xenografts. Effects of different treatment parameters were determined by varying microbubble concentration (0, 8, 80, and 1000 μL/kg), ultrasound pressure (0, 250, 570, and 750 kPa), and radiation doses (0, 2, and 8 Gy). Tumour response monitored after 24 h depicted greater cell death with combined treatment compared to the control or USMB only group. Cell death with USMB only (at 250 kPa) was reported to be 7 ± 2% and for USMB + 8 Gy it was found to be 60 ± 4%. Similarly, USMB only (at 750 kPa) and USMB + 8 Gy demonstrated cell death of 7 ± 2% and 74 ± 5%, respectively. Additionally, the level of ceramide was significantly increased with higher microbubble concentration. The mechanism behind cell death induced by USMB and XRT was reported to be mediated by ceramide. Thus, the study found that varying different parameters of ultrasound, microbubble, and radiation doses caused extensive tumour vasculature destruction and cell death that subsequently coincided with ceramide increase suggesting the involvement of ceramide-signalling pathways in radiation-induced tumour damage [90].

## 6. Clinical Relevance of USMB and Ceramide

To date, no clinical study has been reported that utilized the vascular targeting strategies with radiation to induce ceramide generation for tumour cure. Only one in-human clinical trial has been performed that assessed the safety and feasibility of USMB in clinical settings. The study incorporated 28 patients suffering from hepatocellular carcinoma. Participants were categorized into two groups, the first receiving transarterial radioembolization (TARE) alone and the second one receiving TARE + USMB. The USMB treatment was administered three times after TARE treatment between 1 and 2 weeks. The treatment was reported to have no adverse side effects and was well tolerated by most patients. Tumour response was evaluated in both groups. Results indicated greater tumour response to TARE + USMB treatment in participants compared to TARE treatment alone [69]. In this case, TARE is not purely radiation but an embolic treatment that also causes vascular destruction by blocking blood flow. More such clinical studies should be performed focusing on the mechanism of USMB. To that end, work is undergoing presently with external beam radiotherapy in breast cancer patients and head and neck cancer patients with combined USMB and XRT treatments (NCT04431674 and NCT04431648).

There have been few clinical studies that have looked into the effect of combining USMB with radiation therapy; however, the in-depth mechanism of how both these entities function to elicit tumour response is still a question. The approach of combining USMB with low radiation doses to induce ceramide release has still not been explored clinically. Few groups have utilized high-dose radiation therapy to stimulate ceramide, which has shown promising outcomes in overall tumour control. A study conducted by Sathishkumar and colleagues explored the involvement of ASMase/ceramide in patients with malignant tumours. Each patient was exposed to spatially fractionated high dose (Grid) radiation treatment (SFGRT) and their blood sample was collected to determine the serum ceramide content. The study found that 67% of patients categorized as complete and partial responders demonstrated an elevated level of ceramide content in their serum. On the contrary, no increase in serum ceramide level was found for non-responders. A similar pattern of increase in secretory SMase (S-SMase) was reported in patients that responded completely or partially to SFGRT. The endothelial cells obtained from patients demonstrated higher apoptosis when exposed to SFGRT, suggesting ceramide mediates the radiation response of endothelial cells [98]. Nagahashi et al. explored the role of ceramide and its various metabolites in cancer cell progression in breast patients. Malignant and non-malignant breast tissue lesions from patients were collected for quantitative analysis. The level of ceramide and its metabolites, specifically S1P was found to be higher in the tissue of cancerous breast patients [99]. More specifically, an elevated S1P level is strongly correlated with lymphatic metastasis in breast cancer [100]. Normal breast tissue depicted non-significant changes in the level of various sphingolipids. The total level of ceramide and its various species, namely C14:0, C16:0, C18:1, C18:0, C20:0, C22:0, C24:1, C24:0, C26:1, and C26:0, were found to be elevated significantly in malignant breast tissue [101]. Compared to normal breast tissue, malignant tissue depicted a 12-fold higher ceramide content [102]. Ceramide generated in malignant breast tissue appears to have taken place through activation of all three ceramide biosynthesis pathways [99]. The increased level of ceramide in breast patients is known to be a result of longevity assurance (LASS) genes up-regulation [102]. These genes are known to have an inhibitory effect on cancer cell growth [103]. Taken together, these studies suggested that the enhanced level of ceramide and its metabolites are closely associated with breast cancer progression. The role of ceramide has also been explored in patients with pulmonary and hepatic metastases. Patients receiving stereotactic body radiation therapy (SBRT) and chemotherapy demonstrated a significant-high increase in plasma ceramide level that resulted in complete tumour regression, whereas low ceramide level was associated with increased tumour size. Thus, the study suggested that ceramide can be used as a biomarker for liver and lung oligometastases of colorectal cancer [104]. Similarly, in advanced/metastatic pancreatic adenocarcinoma patients, higher plasma S1P concentration was found to be a favorable prognostic/predictive marker [105]. The role of sphingolipids and their metabolites in the progression and treatment of a variety of cancer types has been widely documented; however, their relevance in brain tumours has been less explored and is not yet fully elucidated. Clinical studies have found higher ceramide levels within brain tumours to be linked with a more favorable prognosis, whereas lower levels of ceramide correlated with poor prognostic outcomes. A study by Riboni et al. indicated ceramide to be a major determinant in the progression of human astrocytomas and patient survival. The level of ceramide was found to be significantly lower in glial tumours compared to peritumoral tissue [106]. A comprehensive study performed on human glioma specimens confirmed 5-fold lower ceramide content and 9-fold higher S1P content compared to tissue specimens collected from normal gray matter, suggesting a direct involvement of ceramide and S1P in tumour growth and progression [107]. Despite these studies that determined the involvement of ceramide and its metabolites in cancer progress, there is a lack of understanding of the actual mechanism that regulates this process. More studies should be carried out to understand the link between the low and enhanced level of sphingolipid with tumour resistance to radiotherapy. Studies combining radiation with USMB might have clinical benefits as the radiation dose can be minimized when using it with USMB. Ceramide induced by a combined treatment of low radiation dose and USMB might be a fruitful approach for cancer cure.

## 7. Conclusions

The present review summarizes the findings that combined USMB and radiation in tumour vascular damage. Upon ultrasound stimulation, microbubbles undergo cavitation inducing bioeffects in tumour microvasculature. The combination of USMB and radiation induces endothelial membrane perturbation, a process known as sonoporation resulting in enhanced vascular permeability and greater vascular damage, appearing to be vascular disruption. USMB and radiation-mediated tumour damage is known to be ceramide dependent. The amount of ceramide required for inducing tumour vascular damage can be induced either by using single high dose radiation (>8–10 Gy) or USMB combined with low radiation doses (2 Gy). Significant damage to endothelial cells leading to vascular collapse and tumour cell death have been reported using USMB and radiation. A Gene profiling study carried out in vitro on an endothelial model has indicated up-regulation of certain genes including sphingomyelinase (SMPD2), UGT8, cytochrome c oxidase (COX6B1), caspase 9, and MAP2K1 following USMB and XRT. These genes are known to code for proteins related to ceramide apoptotic signalling pathways. Similarly, genetic manipulation of UGT8 in vivo by downregulating this particular gene resulted in a higher level of ceramide generation, which caused significant tumour damage and diminishment of blood flow and oxygen saturation upon exposure to USMB and XRT. The up-regulation of UGT8 exhibited lower intracellular ceramide levels with no significant tumour vasculature damage. These findings have only been validated in in vitro and in vivo studies; however, the findings still need to be recapitulated in clinical settings. Research is underway to monitor tumour response either by using USMB combined with high radiation doses or by using ceramide as a modulator for various cellular processes. More clinical trials should be conducted utilizing the combined approach of USMB and radiation in ceramide-induced tumour response. Emerging clinical data have indicated sphingolipid metabolites to be a good predictive marker for tumour progression and growth. The level of ceramide is found to be lower in different cancer types, as opposed to S1P, which is usually up-regulated and promotes tumour growth, mediating radioresistance in tumours. Clinical studies should be designed that focus on activating ceramide signalling pathways in radioresistant tumours. Accurate and advanced methods/technologies should be incorporated to determine the levels of ceramide and S1P in the tumour microenvironment; this will help in designing targeted therapies against ceramide and S1P signalling for treating cancer patients.

## Data Availability

Not applicable.
